# Predictive processing in time perception: Assessing prediction error minimization in the sub-second range

**DOI:** 10.3758/s13415-026-01471-9

**Published:** 2026-06-24

**Authors:** Maki Uraguchi, Hideki Ohira

**Affiliations:** Department of Psychology, Graduate School of Informatics, Nagoya University, Furo-Cho, Chikusa-Ku, Nagoya, 464-8601 Japan

**Keywords:** Time perception, Predictive processing, Prediction error minimization, Prediction updating, Temporal generalization

## Abstract

The predictive processing theory proposes that minimization of prediction error is a governing principle of perception, learning, and behavior, yet few studies have directly tested this hypothesis in time perception. We operationalized prediction error minimization as updating of an internal temporal reference and examined whether reference updating follows the direction required to reduce systematic prediction error. Across two experiments, 60 university students (Experiment 1) and 359 adults from a broader age range (Experiment 2) performed a temporal generalization task. Reference updating was assessed from changes in distributional asymmetry and peak shifts of temporal generalization gradients. This dissociated reference updating from possible perceptual bias that would alter gradient steepness without shifting the peak. In Experiment 1, frequent presentation of longer or shorter durations produced expected directional shifts of the internal temporal reference. In Experiment 2, consistent directional shifts emerged only when prediction error was sufficiently large, whereas smaller prediction error failed to induce reliable shifts. Together, these findings demonstrate that internal temporal reference updating in sub-second range time perception depends on the direction and magnitude of prediction error, providing a direct behavioral test of the prediction error minimization hypothesis.

The theory of predictive processing has gained increasing attention in time perception research over the last decade. Predictive processing suggests that the brain constantly generates and updates predictions about incoming sensory information and aims to minimize the difference between the predictions and actual sensory inputs (prediction errors). Researchers across various disciplines, including neuroscience, cognitive science, psychology, and philosophy, have offered perspectives on its application and extension across different research domains. These contributions collectively enhance our understanding of how the brain generates predictions, integrates sensory information, and guides adaptive behavior (Barrett, 2017; Barrett & Simmons, 2015; Clark, 2013, 2015; Friston et al., 2006; Friston, 2005, 2010; Hohwy, 2013; Rao & Ballard, 1999).

A growing body of empirical and computational work in humans and animals suggests that predictive processing provides a plausible framework for understanding time perception. Shi and Burr (2016) suggested that subjective time with context effects arose from prediction error minimization and prediction updating within the predictive coding framework. Sadibolova and Terhune (2022) discussed predictive processing related to Bayesian models, particularly in likelihood conceptualization. Several empirical studies have interpreted observed phenomena or experimental results within a predictive processing framework (Clarke & Tyler, 2024; Jalalkamali & Nazari, 2023; Schweitzer et al., 2017; Sherman et al., 2022). Fountas et al. (2022) proposed a computational model grounded in the predictive processing framework. This model generated subjective duration estimates, which closely mirrored human reports from more than 12,000 participants. Meirhaeghe et al. (2021) provided compelling evidence that the brain updated predictions based on environmental statistics and used these predictions to modulate sensory processing, a core tenet of the predictive processing theory. They used a time reproduction task (the "Ready-Set-Go" paradigm) in non-human primates to demonstrate that the speed of neural dynamics in the dorsomedial frontal cortex (DMFC) adjusted according to the average timing of the expected stimuli. This adjustment, termed temporal scaling, allowed neural activity to align with the statistical mean of the stimulus intervals, effectively encoding time relative to expectations. Moreover, when the temporal statistics of the stimuli changed covertly, neural responses adapted predictably to reflect the new mean timing, which indicated a dynamic updating of internal predictions. This dual process, updating predictions based on new temporal statistics and modulating neural dynamics to align with these predictions, illustrates the full scope of predictive processing in time perception. These studies support the plausibility of predictive processing accounts in time perception, while leaving open the question of how one can directly test its core hypothesis—prediction error minimization—at the behavioral level.

Prediction error minimization is the central hypothesis of predictive processing, according to which perceptual bias, action, and learning constitute complementary strategies for reducing prediction error. Experience-dependent updating of internal representations has been extensively documented within foundational frameworks for Bayesian learning and sensorimotor control (Acerbi et al., 2012; Berniker et al., 2010; Kawato et al., 1987; Körding & Wolpert, 2004; Körding et al., 2007; Miyazaki et al., 2005). In these approaches, adaptive behavior is typically characterized in terms of statistically optimal or near-optimal inference, within which the reduction of prediction error is implicit rather than treated as an independently testable principle. In time perception research, Bayesian modeling studies have shown that contextual effects such as regression to the mean and time-order errors can be explained by perceptual biases arising from latent priors shaped by task structure (Burr et al., 2013; Cicchini et al., 2012; de Jong et al., 2021; Glasauer & Shi, 2021; Jazayeri & Shadlen, 2010), and several studies have examined how temporal priors are formed and updated (Roach et al., 2017). However, while this body of work establishes that priors influence temporal judgments and can be learned from experience, it has not explicitly tested prediction error minimization as a governing principle of reference updating—namely, whether updating follows the direction required to reduce systematic prediction error—because this principle is typically assumed rather than evaluated.

The present study addresses this gap by providing a direct behavioral test of the prediction error minimization hypothesis in time perception in the sub-second range. We operationalize prediction error minimization as updating of the internal temporal reference in a manner that reduces systematic prediction error. To test this empirically, it is necessary to rely on a behavioral indicator that does not presuppose Bayesian updating and that can dissociate reference updating from perceptual bias. To implement this diagnostic logic behaviorally, we employ the temporal generalization task.

The temporal generalization task, a single-stimulus method (Grondin, 2010), has been widely used in time-perception research with both animals and humans across sub-second and supra-second durations. In the present study, we focused on the conventional temporal generalization task used in human research (Wearden, 1992; Wearden & Towse, 1994). At the beginning of the task, participants were instructed to memorize a standard duration. Subsequently, comparison stimuli were presented individually, and participants judged whether each comparison had a duration identical to the memorized standard. Responses are summarized as a temporal generalization gradient, which indicates the proportion of “same” responses for each comparison duration. It is important to note that this task does not aim to measure the perceived duration of each comparison stimulus; rather, it investigates the nature of internal temporal representations through generalization across comparison durations.

The judgment required in this task entails comparing the memorized standard with each presented comparison stimulus. In this process, the internal temporal reference functions as a prediction about stimulus duration, which is compared with the incoming sensory input. Importantly, such a reference need not be maintained solely as an explicit memory trace. Previous work has shown that observers may rely on implicitly inferred references derived from the statistical structure of the presented stimuli (Morgan et al., 2000, 2012; Nachmias, 2006) and that such implicit statistical learning can also support the formation of temporal references in time perception tasks (Otsuka et al., 2025). Regardless of how the reference is constructed, temporal judgments in the generalization task necessarily involve a comparison between an expected duration and the presented stimulus, thereby generating a prediction error signal.

The shape of the temporal generalization gradient allows two distinct signatures to be dissociated: biased perception of the presented stimuli and updating of the internal temporal reference. These two processes can be understood as alternative or concurrent ways of reducing prediction error generated during temporal judgment. When only perceptual bias is present—most plausibly in the form of regression toward the mean—individual comparison durations may be perceived as closer to the standard than they physically are. Being more similar to the standard under this biased perception, comparison stimuli are therefore more likely to be judged as the same, increasing the proportion of “same” responses across a range of durations. This effect results in a flatter or less steep gradient, but it does not produce a systematic shift in the location of the gradient peak. In contrast, when the internal temporal reference itself is updated to be longer or shorter, the distribution of “same” responses changes asymmetrically toward longer or shorter comparison durations. If the internal reference shifts toward longer durations, longer comparison stimuli receive a higher proportion of “same” responses, yielding a skewed gradient accompanied by a shift of the peak. In practice, both perceptual bias and reference updating may contribute to the observed gradient shape. Nevertheless, because perceptual bias alone does not predict a systematic peak shift, analyses focusing on gradient skewness and peak location provide a principled means of assessing updating of the internal temporal reference, which is the focus of the present study.

Based on these considerations, we formulated the following hypotheses. If prediction updating in the temporal generalization task reflects prediction error minimization, the direction of updating should follow the direction required to reduce systematic prediction error. Accordingly, frequent presentation of longer comparison durations should shift the internal temporal reference toward longer values, whereas frequent presentation of shorter comparison durations should shift it toward shorter values. Furthermore, once the internal reference has shifted, subsequent presentation of comparison stimuli with a uniform frequency should update the reference toward the mean of the comparison distribution. This updating is expected to manifest as asymmetric changes in the temporal generalization gradient accompanied by a shift of its peak. To quantify these changes, we analyzed both the distributional asymmetry and the peak location of the generalization gradient. Specifically, we computed the subjective standard value (SSV) as the same-response–weighted mean of comparison durations (Klapproth & Wearden, 2011), which provides a summary index of distributional asymmetry of the gradient, and applied Gaussian fitting to estimate the peak location of the gradient. Converging evidence from these two indices was interpreted as evidence of updating of the internal temporal reference. Finally, we compared logarithmically and linearly spaced comparison sets to examine whether different stimulus structures produce distinct patterns of prediction updating. In addition, because Gaussian fitting is less sensitive to truncation of the gradient at the boundaries of the stimulus range and can be applied to smaller trial windows, we conducted exploratory block-level analyses of Gaussian *μ* to descriptively examine early reference-updating dynamics.

## Experiment 1

### Methods

#### Participants

The final analyzed sample comprised 60 healthy right-handed undergraduate and graduate students from universities in Nagoya City (30 men, 30 women; age range: 18–24 years; mean age = 20.01 years). All participants provided written and oral informed consent, and none reported hearing difficulties.

Sample size was determined based on the effect size (*η*^*2*^ = .263, converted to* f*^*2*^ = .357) derived from the peak-shift effect reported by Bizo and McMahon (2007), *F*(2, 39) = 6.97. A power analysis conducted in R (pwr package, version 1.2–2; Champely et al., 2018) indicated that 39 participants would be sufficient to achieve .80 power at *α* = .05. To ensure adequate power and allow for exclusions, we recruited 30 participants per group.

During recruitment-level screening, five individuals were excluded based on post-experiment questionnaires (four reported current medication use; one reported left-handedness). Additional participants were recruited until the target sample size of eligible participants was reached.

#### Experimental design

A between-subjects design was used. Participants were randomly assigned to either a long-condition group or a short-condition group. During the skewed distribution phase, the longest comparison stimulus was overrepresented in the long-condition group, whereas the shortest comparison stimulus was overrepresented in the short-condition group.

#### Temporal generalization task

##### Stimuli

Auditory stimuli were 500-Hz pure tones. In the main task, the standard stimulus duration was 600 ms. Comparison stimuli were 425, 476, 535, 600, 673, 755, and 847 ms. Stimuli were generated in MATLAB (R2018a; The MathWorks, Inc., Natick, MA) at a 44,100-Hz sampling rate and shaped with 5-ms cosine onset and offset ramps.

##### Practice session

Before the main task, participants completed a brief practice session to familiarize them with the trial structure and response mapping. They were explicitly informed that the stimulus durations used during practice would differ from those used in the main experiment. Practice stimuli were 500-Hz pure tones with a 400-ms standard and comparison durations of 100, 200, 300, 400, 500, 600, and 700 ms. Participants were instructed to memorize the standard duration and judge whether each comparison duration was the same as the memorized standard. After one guided trial, participants completed the full practice procedure, consisting of standard presentations followed by seven judgment trials (one per comparison duration). Response mapping was counterbalanced across participants and held constant between practice and main tasks. No feedback was provided.

All participants completed the same practice procedure, which was intended solely for familiarization with the task structure and response requirements and not to induce learning or conditioning. The experimenter remained present, and practice was repeated if participants requested additional familiarization.

##### Main task procedure

At the start of the main task, a fixation cross was presented. The first standard tone was delivered 2.6 s after fixation onset, followed by four additional presentations of the 600-ms standard (interstimulus intervals: 2.1–2.8 s). Judgment trials then began seamlessly. On each trial, a question mark appeared, followed after 1.8–2.5 s by a single comparison tone. Participants judged whether the tone was the same as the memorized standard using the assigned arrow key. The next trial began 1.5 s after the response. No feedback was provided.

**Fig. 1:**
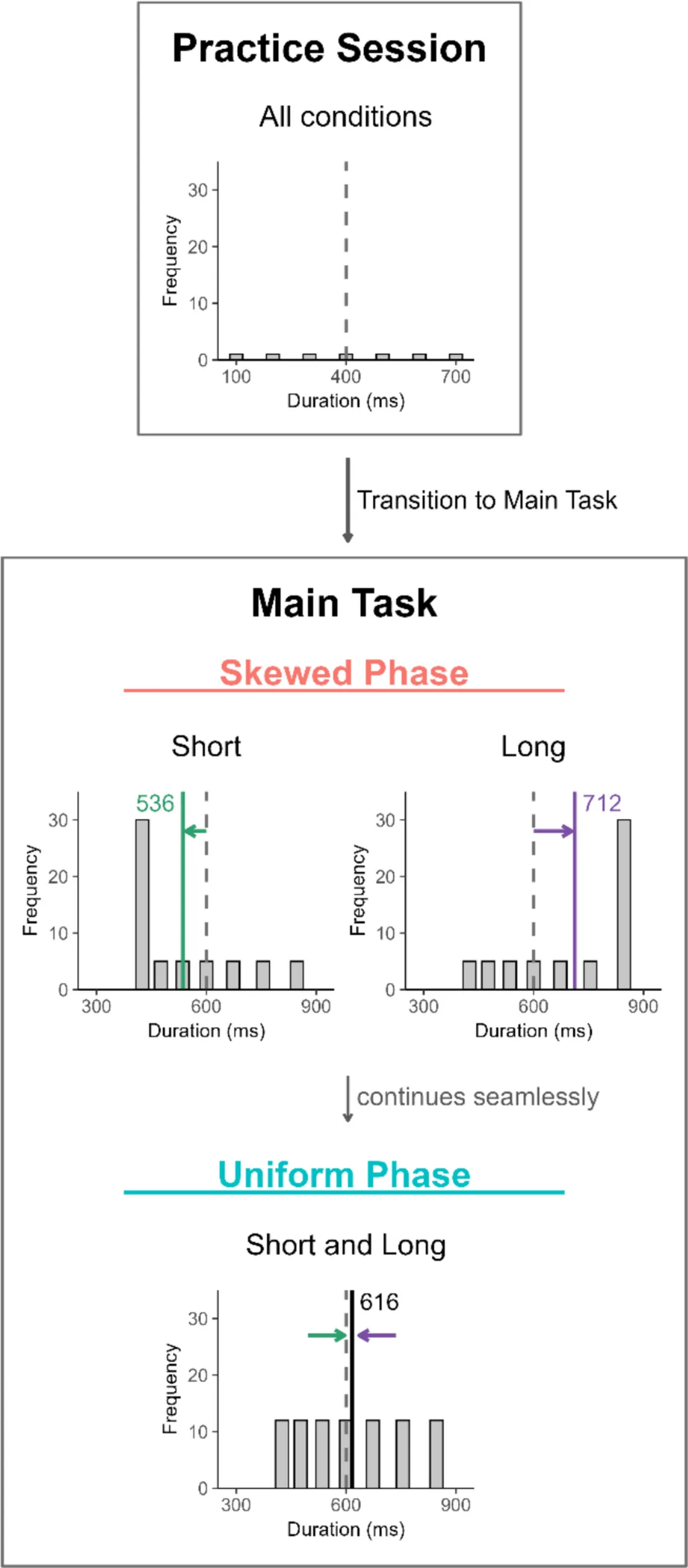
Experimental structure and stimulus distributions in Experiment 1. All participants completed the Practice Session for familiarization using a 400-ms standard duration and comparison durations ranging from 100 to 700 ms. In the Main Task, comparison stimuli were logarithmically spaced around a 600-ms standard duration. In the Skewed Phase, either the shortest or longest comparison stimulus was presented more frequently, depending on condition. In the subsequent Uniform Phase, all comparison stimuli were presented equally often. Dashed vertical lines indicate the standard duration. Colored vertical lines (purple: Long condition; green: Short condition) indicate the weighted mean of the presented comparison-duration distribution in the Skewed Phase. In the Uniform Phase, the black vertical line indicates the weighted mean of the uniform stimulus distribution. Colored arrows illustrate the expected directional shift of internal predictions toward reducing prediction error based on the stimulus distribution in each phase and condition

The task consisted of two successive phases without a scheduled break: a skewed distribution phase (60 trials) followed by a uniform distribution phase (84 trials). In the skewed distribution phase, either the longest (847 ms) or shortest (425 ms) comparison was presented 30 times, and each remaining duration five times. In the uniform distribution phase, each comparison duration was presented 12 times. Stimuli were presented in pseudorandom order. The full task lasted approximately 15 min. The task structure and stimulus distributions for each phase and condition are illustrated in Fig. 1.

##### Apparatus and software

The experiment was controlled using MATLAB and Psychtoolbox-3 (Brainard, 1997; Kleiner et al., 2007; Pelli, 1997) on Linux Ubuntu 18.04. Auditory stimuli were delivered via an external audio interface (Steinberg UR22mkII) and circumaural headphones (Beyerdynamic DT990 PRO). Visual cues were displayed on an LCD monitor, and responses were collected via a standard keyboard.

#### Data quality checks and exclusion criteria

Data analysis comprised four stages: 1) behavioral screening and exclusion; 2) construction of temporal generalization gradients; 3) derivation of summary and model-based indices of reference location; and 4) inferential statistical testing. Stage 1 is described below; Stages 2 and 3 are described in Data Processing and Dependent Measures, and Stage 4 is described in *Statistical Analysis*.

Behavioral data were screened prior to analysis. No participant showed unrealistically short response latencies (< 150 ms). Participants with excessively long latencies (> 20 s) were excluded (long condition: 1; short condition: 3). No additional exclusions were made based on response-pattern screening (e.g., exclusive use of a single key).

For Gaussian fitting (full-phase), only participants with convergent fits within predefined plausible parameter ranges were included (0 < *μ* < 1000 ms; 0 < *σ* < 500 ms; *R*^*2*^ > .40). For the exploratory block-level Gaussian fitting, only plausibility constraints were applied and convergence across all blocks was required; no *R*^*2*^ threshold was imposed because each block contained substantially fewer trials, which substantially reduces the stability of goodness-of-fit estimates. Final sample sizes for each analysis are reported in Table 1.

**Table 1:** Sample sizes across analyses in Experiment 1

Analysis	N in long condition	N in short condition	Notes
Final analyzed sample	30	30	After recruitment-level screening
After behavioral screening	Skewed phase: 29 Uniform phase: 29	Skewed phase: 30 Uniform phase: 27	Latency > 20 s
Temporal generalization gradients (proportion of “same” responses)	29	27	Paired data only (skewed and uniform phases)
Subjective standard value	29	27	Paired data only (skewed and uniform phases)
Gaussian fitting (full-phase)	25	21	Successful Gaussian fits (paired data; 0 < *μ* < 1000 ms, *σ* < 500 ms, *R*^*2*^ > .40)
Exploratory block-level Gaussian fitting	9	12	Successful Gaussian fits (paired data; 0 < *μ* < 1000 ms, *σ* < 500 ms)

#### Data processing and dependent measures

##### Temporal generalization gradients

For each participant, proportions of “same” responses were calculated for each comparison stimulus separately for the skewed and uniform distribution phases. Group-level gradients were obtained by averaging across participants for visualization.

##### Subjective standard value

Subjective standard value (SSV) was computed following Klapproth and Wearden (2011) as the same-response–weighted mean of comparison durations. Specifically, the sum of the products of each comparison duration and the number of “same” responses to that duration was divided by the total number of “same” responses. Subjective standard value were calculated separately for each phase and each participant.

##### Gaussian fitting

Gaussian functions were fitted to each participant’s stimulus-wise proportions of “same” responses separately for each phase using nonlinear least squares (minpack.lm, version 1.2.4; Elzhov et al., 2024). The mean parameter (*μ*) was extracted as an estimate of peak location. Kernel density estimation (KDE) was applied to *μ* values for descriptive visualization only.

##### Exploratory block-level Gaussian fitting

To examine early updating, Gaussian *μ* was also estimated at the block level (12-trial blocks in the skewed distribution phase; 7-trial blocks in the uniform distribution phase). This analysis was treated as exploratory.

#### Statistical analysis

All analyses were conducted in R (version 4.4.3; R Core Team, 2025). Phase differences in temporal generalization gradients and between-group differences in block-level Gaussian *μ* were assessed by using bootstrap resampling (10,000 samples; Efron & Tibshirani, 1993), implemented with the boot package (version 1.3–28; Canty & Ripley, 2021). Subjective standard value and Gaussian *μ* values estimated from the full phase were analyzed using linear mixed-effects models (LMMs; lme4; Bates et al., 2015) with Type III tests (lmerTest; Kuznetsova et al., 2017). Partial *R*^*2*^ values were computed using r2glmm (Jaeger et al., 2017). Post hoc comparisons were conducted with emmeans (version 2.0.1; Lenth & Piaskowski, 2025) by using Bonferroni-adjusted *p* values.

### Results

#### Temporal generalization gradients: Distributional asymmetry

**Fig. 2:**
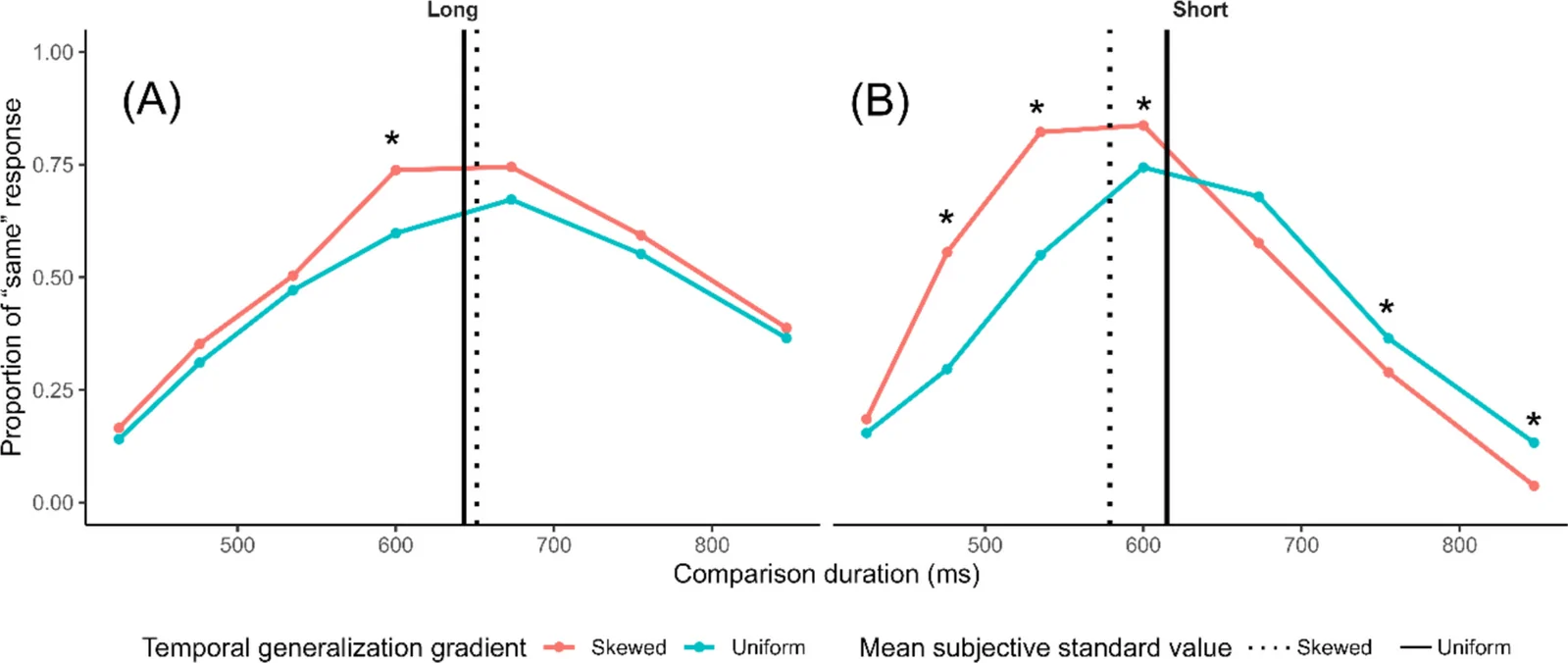
Temporal generalization gradients across phases in Experiment 1. (**A**) Long condition. (**B**) Short condition. Group mean proportions of “same” responses are shown as a function of comparison duration for the Skewed and Uniform phases. Asterisks indicate statistically significant phase differences at corresponding comparison durations (*p* < .05). Dotted and solid vertical lines represent the group mean subjective standard values (SSVs) in the Skewed and Uniform phases, respectively

Figure 2 summarizes the group-mean temporal generalization gradients across phases. Vertical lines indicate the corresponding mean SSVs.

##### Long-condition group (*n* = 29)

Bootstrap resampling revealed a statistically reliable reduction in the proportion of “same” responses in the uniform relative to the skewed distribution phase at the 600-ms comparison stimulus (*estimate* =  − 0.140, 95% CI [− 0.239, − 0.044]). No other comparison stimuli showed statistically reliable phase differences.

##### Short-condition group (*n* = 27)

Bootstrap resampling revealed statistically reliable phase differences across multiple comparison stimuli. Relative to the skewed distribution phase, the uniform distribution phase showed reduced “same” responses for shorter-than-standard durations (e.g., 476 ms: *estimate* =  − 0.259, 95% CI [− 0.375, − 0.144]; 535 ms: *estimate* =  − 0.273, 95% CI [− 0.385, − 0.156]) and increased “same” responses for longer-than-standard durations (e.g., 755 ms: *estimate* = 0.075, 95% CI [0.009, 0.149]; 847 ms: *estimate* = 0.096, 95% CI [0.032, 0.175]). No statistically reliable difference was observed near the standard (673 ms). This pattern indicates a phase-dependent change in distributional asymmetry.

#### Subjective standard value

Distributional asymmetry was summarized using subjective standard values (SSVs). For the long-condition group (*n* = 29), mean SSVs (± *SD*) were 651.23 ms (70.78) and 642.95 ms (78.29) in the skewed and uniform distribution phases, respectively; for the short-condition group (*n* = 27), the corresponding mean SSVs were 578.75 ms (40.95) and 614.82 ms (55.80). The corresponding mean SSVs are illustrated by the vertical lines in Fig. 2.

Changes in SSV were analyzed using a LMM with Group (long vs. short) and Phase (skewed vs. uniform) as fixed effects and participant as a random intercept. The model revealed significant main effects of Group, *F*(1, 54) = 9.06, *p* = .004, and Phase, *F*(1, 54) = 9.47, *p* = .003, as well as a significant Group × Phase interaction, *F*(1, 54) = 24.11, *p* < .001. The interaction accounted for the largest proportion of unique variance (partial *R*^*2*^ = .309, 95% CI [.134, .499]), followed by Phase (partial *R*^*2*^ = .149, 95% CI [.022, .342]) and Group (partial *R*^*2*^ = .144, 95% CI [.020, .336]); the fixed-effects model explained 36.2% of the variance (model *R*^*2*^ = .362, 95% CI [.219, .528]).

**Fig. 3:**
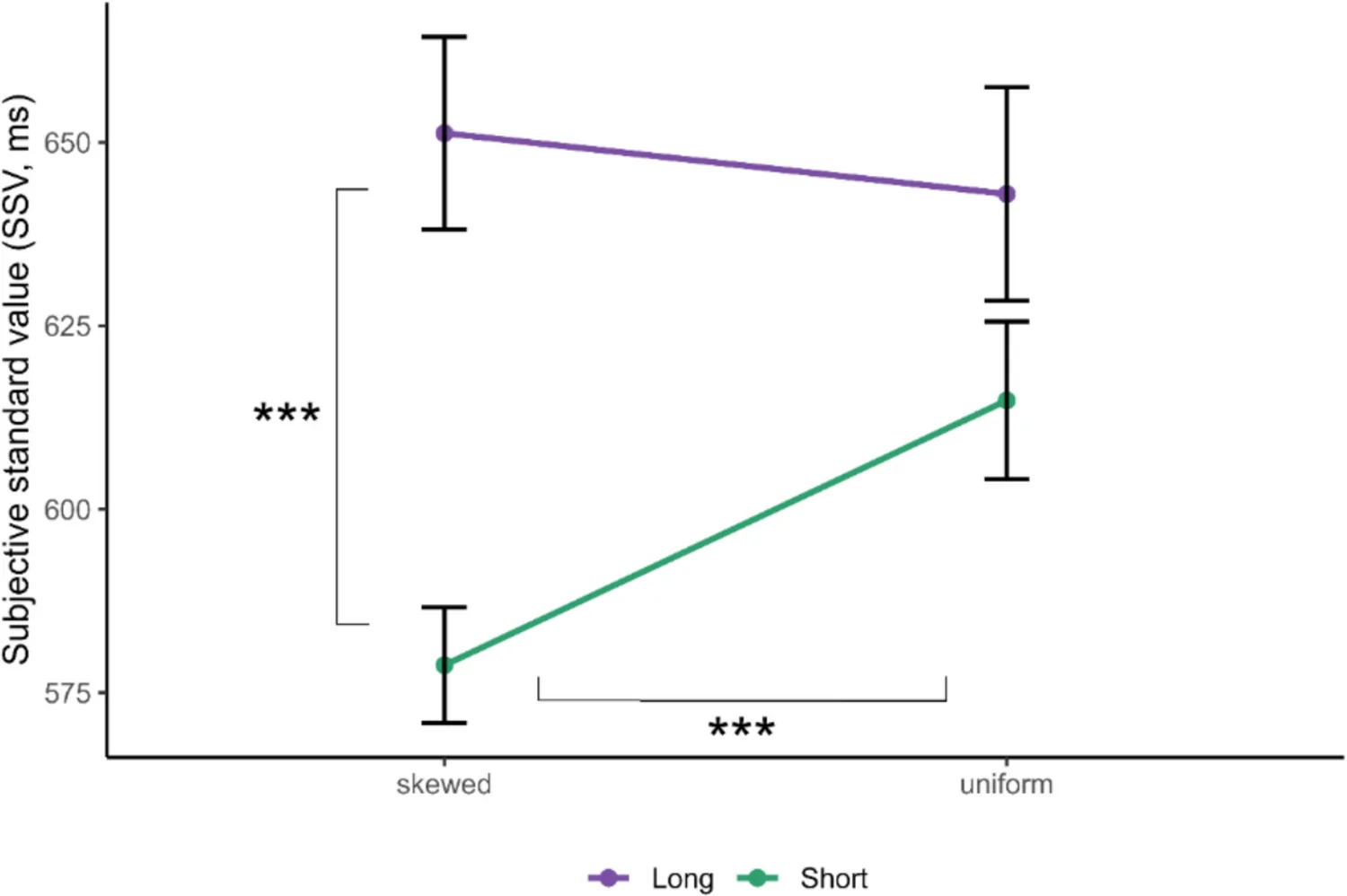
Phase-dependent changes in subjective standard values in Experiment 1. Group mean subjective standard values (SSVs) are shown for the skewed and uniform distribution phases in the Long and Short conditions. Results are based on a linear mixed-effects model. Error bars represent ± 1 *SE*. Asterisks indicate statistically significant contrasts (***p < .001)

Post hoc comparisons (skewed − uniform) showed a significant increase in SSV from the skewed to the uniform distribution phase in the short-condition group (*estimate* =  − 36.07 ms, *SE* = 6.50, *p* < .0001), whereas no significant phase-related change was observed in the long-condition group (*estimate* = 8.28 ms, *SE* = 6.27, *p* = .19). During the skewed distribution phase, SSV was significantly higher in the long- than in the short-condition group (*estimate* = 72.5 ms, *SE* = 17.3, *p* = .0001); this group difference was not significant in the uniform distribution phase (*estimate* = 28.1 ms, *SE* = 17.3, *p* = .11). Figure 3 presents the LMM results for SSV.

#### Gaussian fit of generalization gradients: Peak shift

**Fig. 4:**
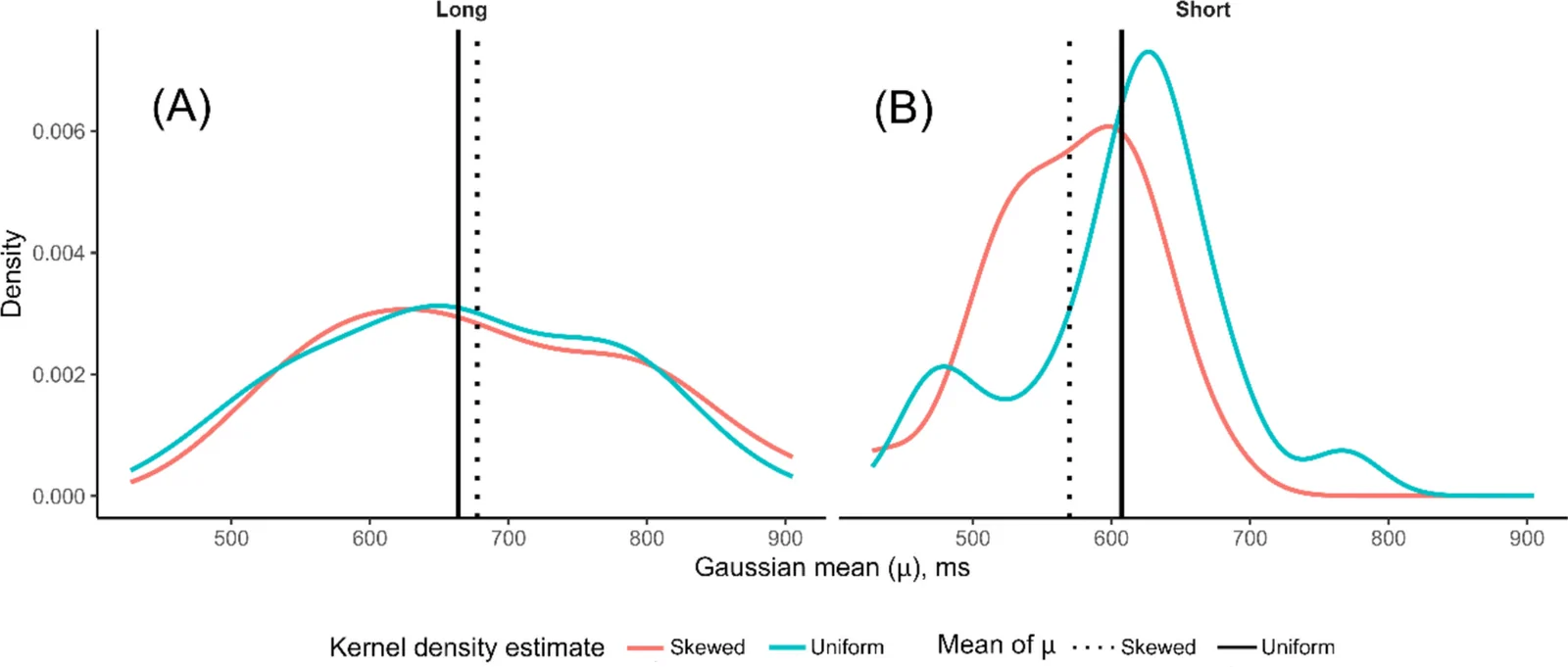
Distribution of participant-level Gaussian *μ* estimates across phases in Experiment 1. (**A**) Long condition. (**B**) Short condition. Kernel density estimates depict the distribution of Gaussian mean (*μ*) values obtained by fitting Gaussian functions to each participant’s temporal generalization gradients in the Skewed and Uniform phases. Dotted and solid vertical lines represent the phase-wise mean *μ* values in the Skewed and Uniform phases, respectively

Because SSVs can be influenced by truncation of the stimulus range, Gaussian fitting was used to estimate the peak location of the internal temporal reference. For the long-condition group (*n* = 25), mean Gaussian *μ* values were 677.56 ms and 663.82 ms in the skewed and uniform distribution phases, respectively; for the short-condition group (*n* = 21), the corresponding mean *μ* values were 569.81 ms and 607.15 ms. Figure 4 shows the distributions of participant-level Gaussian *μ* estimates across phases, with vertical lines indicating the phase-wise mean *μ*.

Gaussian *μ* estimates were analyzed using a LMM with Group and Phase as fixed effects and participant as a random intercept. The model revealed a significant main effect of Group, *F*(1, 44) = 9.89, *p* = .003, a nonsignificant main effect of Phase, *F*(1, 44) = 3.02, *p* = .089, and a significant Group × Phase interaction, *F*(1, 44) = 14.16, *p* < .001. The interaction accounted for the largest proportion of unique variance (partial *R*^*2*^ = .243, 95% CI [.067, .460]), followed by Group (partial *R*^*2*^ = .184, 95% CI [.030, .401]), whereas the contribution of Phase was comparatively small (partial *R*^*2*^ = .064, 95% CI [0, .255]); the fixed-effects model explained 30.4% of the variance in *μ* (model *R*^*2*^ = .304, 95% CI [.154, .498]).

**Fig. 5:**
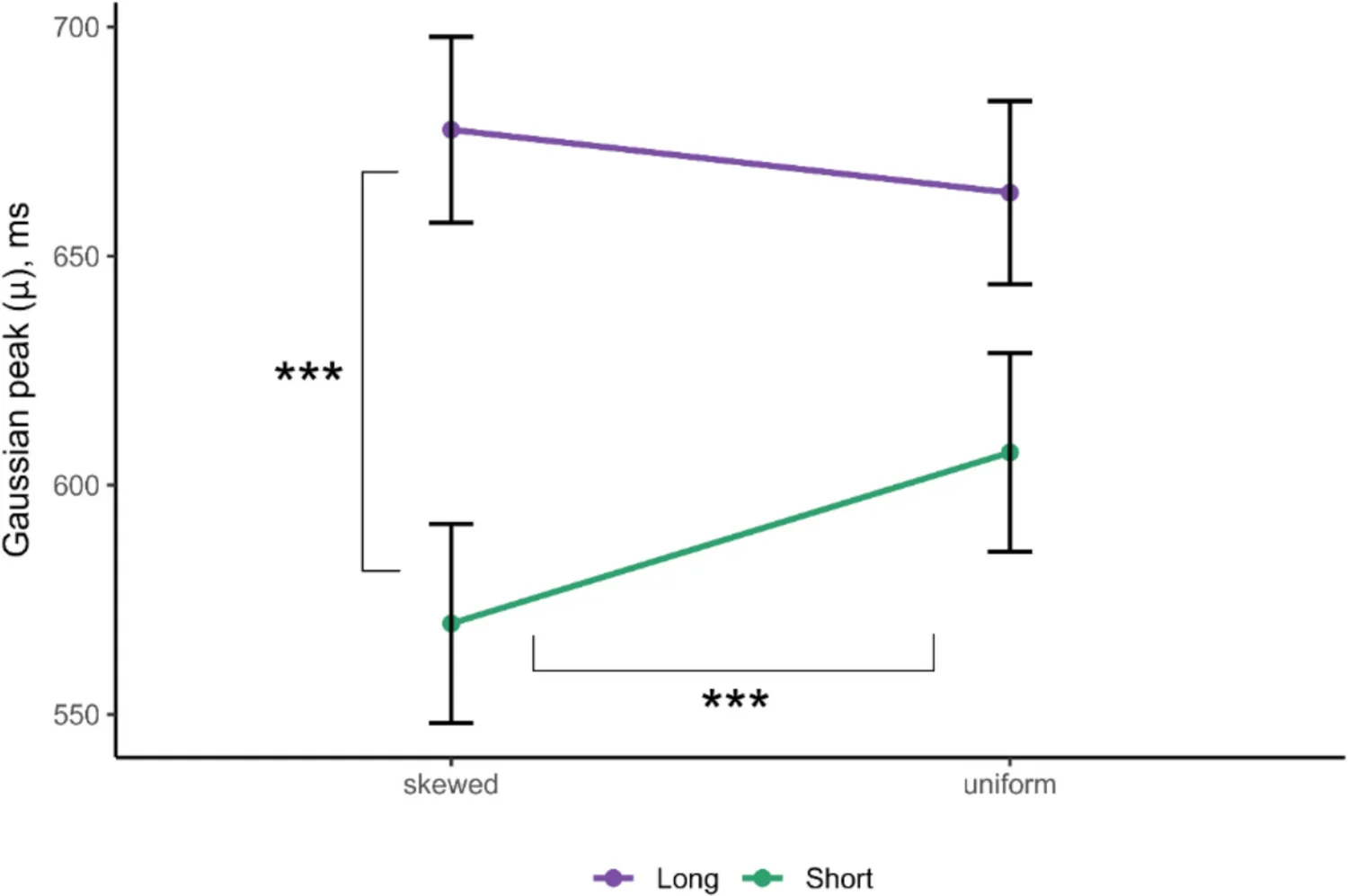
Phase-dependent shifts in Gaussian peak location (*μ*) in Experiment 1. Group mean Gaussian peak locations (*μ*) are shown for the skewed and uniform distribution phases in the Long and Short conditions. Results are based on a linear mixed-effects model. Error bars represent ± 1 *SE*. Asterisks indicate statistically significant contrasts (***p < .001)

Post hoc comparisons (skewed − uniform) showed a significant increase in *μ* from the skewed to the uniform distribution phase in the short-condition group (*estimate* =  − 37.3 ms, *SE* = 10, *p* = .0005), whereas no significant phase-related change was observed in the long-condition group (*estimate* = 13.7 ms, *SE* = 9.17, *p* = .14). During the skewed distribution phase, *μ* was significantly higher in the long- than in the short-condition group (*estimate* = 107.8 ms, *SE* = 27.0, *p* = .0002); although this group difference remained significant in the uniform distribution phase (*estimate* = 56.7 ms, *SE* = 27.0, *p* = .041), it was substantially reduced. Figure 5 presents the LMM results for *μ*.

#### Exploratory block-level Gaussian fit: Early reference updating

To examine whether directional updating emerged early in learning, Gaussian peak locations (*μ*) were estimated separately for successive blocks within each phase and compared between groups (long − short). This exploratory analysis was restricted to participants whose Gaussian fits converged across all blocks and therefore serves to characterize temporal patterns rather than provide confirmatory tests.

During the skewed distribution phase, a reliable separation between groups was already evident in the first block and persisted across subsequent blocks, with higher *μ* values in the long-condition group than in the short-condition group. This pattern indicates that directional displacement of the generalization peak emerged rapidly under skewed input statistics and continued to evolve across early exposure.

**Fig. 6:**
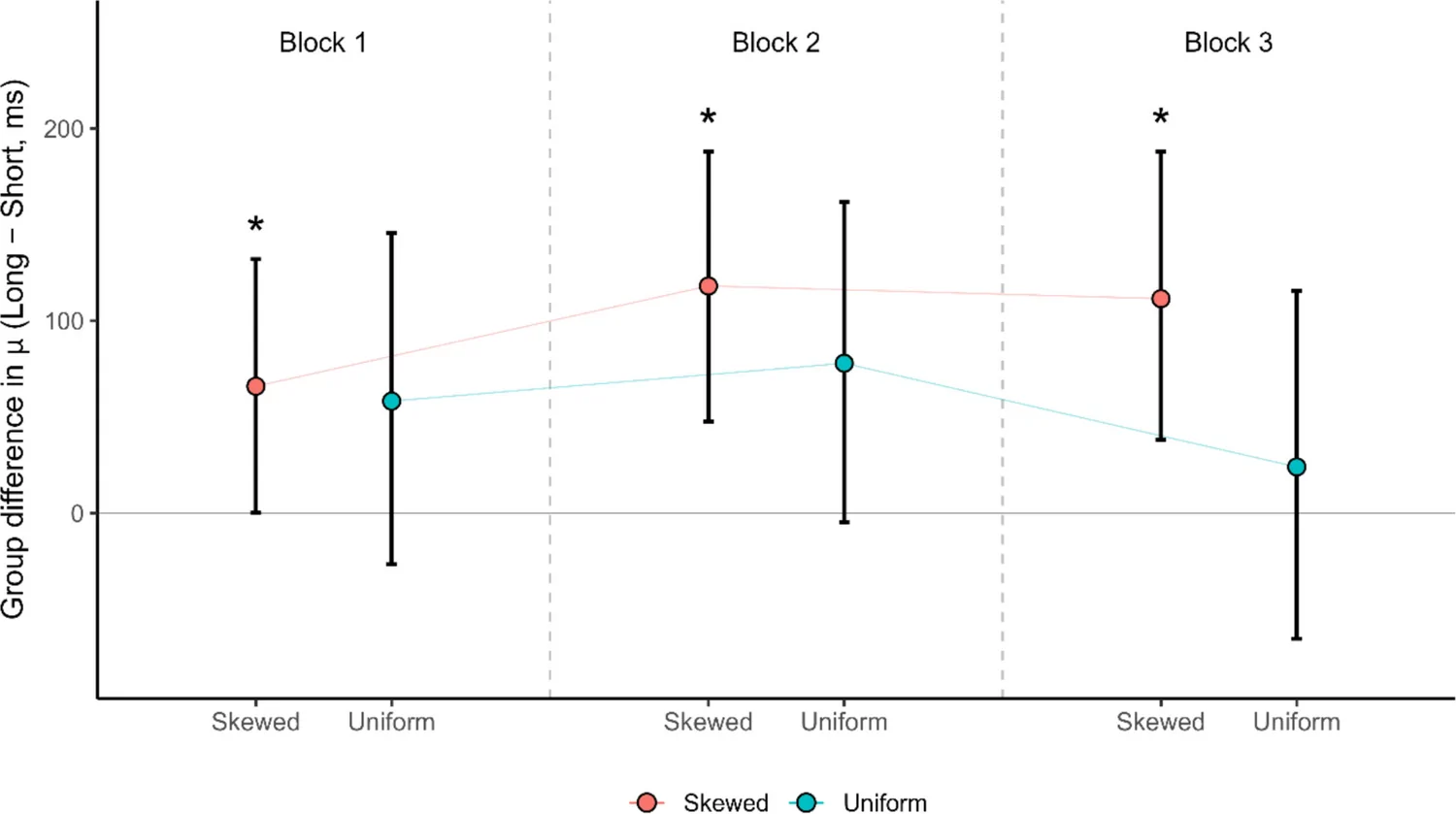
Block-level group differences in Gaussian peak location (*μ*) in Experiment 1. Within each block, bootstrapped estimates of the group difference in Gaussian *μ* (Long − Short) are shown separately for the skewed and uniform phases. Error bars indicate 95% bootstrap confidence intervals. The gray horizontal solid line marks zero difference. Asterisks indicate intervals that do not include zero, reflecting statistically reliable group differences. Reliable between-group differences were observed across blocks during the skewed phase but not during the uniform phase, consistent with early directional updating in each phase

In the uniform distribution phase, between-group differences were no longer statistically reliable at any block, and the point estimates were smaller in the third block than in the preceding blocks, consistent with convergence toward a common reference value. Together, these exploratory results suggest that reference updating began early in both the skewed and uniform phases. Block-level group differences in Gaussian peak location are shown in Fig. 6.

### Discussion

This experiment examined whether the direction of temporal reference updating conforms to the prediction error minimization hypothesis. Using a temporal generalization task, we assessed reference updating from distributional asymmetry and shifts in the peak location of the generalization gradient. We hypothesized that skewed stimulus distributions would induce directional displacement of the internal temporal reference in the direction of prediction error and that subsequent exposure to a uniform distribution would update the reference toward the mean of the comparison set.

Across converging indices, the results supported these hypotheses during the skewed distribution phase: both SSVs and Gaussian peak locations (*μ*) showed clear directional updating. During the uniform distribution phase, however, reliable updating toward the mean was observed only in the short-condition group, whereas no statistically reliable updating was detected in the long-condition group. This asymmetry was also evident in the kernel density estimates of *μ*, while exploratory block-level analyses indicated that reference updating can emerge early within both phases. Together, these results indicate that expected updating was evident under skewed distributions in both groups but was attenuated in the long-condition group during the uniform distribution phase.

One interpretation of this asymmetric outcome is that it arises from the distributional structure of the comparison stimuli, which were logarithmically spaced around the standard. The longer comparisons (673, 755, and 847 ms) deviated more from the standard (600 ms) than the shorter ones (535, 476, and 425 ms), generating asymmetric prediction error. Under this account, the absence of reliable updating in the long-condition group during the uniform phase may reflect insufficient destabilization of the long-shifted reference. This interpretation directly motivated Experiment 2, which tested whether reducing this asymmetry using linearly spaced stimuli would promote reference updating in the long-condition group.

Several additional factors may also have influenced the sensitivity of the present experiment. First, the sample size may have limited power to detect modest shifts in reference updating. Second, the predominantly young adult sample may have been particularly sensitive to contextual influences. Third, the standard and comparison durations presented during the practice session may have unintentionally shaped initial temporal expectations. Although practice was intended only to familiarize participants with the procedure, its potential influence cannot be entirely ruled out, a concern particularly relevant within predictive processing accounts emphasizing the role of prior experience. Finally, the use of arrow keys located on the right side of the keyboard may have introduced subtle motor or attentional asymmetries. Although no single factor can account for the observed pattern, they underscore the lability of time perception and its susceptibility to contextual and procedural influences (Matthews & Meck, 2014). Accordingly, Experiment 2 was designed to directly test whether the asymmetric results observed in Experiment 1 were driven by the asymmetric prediction error induced by logarithmically spaced stimuli, while minimizing potential sources of unintended influence.

## Experiment 2

### Methods

#### Participants

The final analyzed sample comprised 359 registered workers recruited via CrowdWorks, Inc., a large Japanese crowdsourcing platform (180 men, 179 women; age range: 18–78 years; mean age = 43.31 years). All participants provided informed consent before participation.

Based on the results of Experiment 1, sample size planning assumed a smaller effect. Power analysis using the pwr package in R indicated that 128 participants (64 per group) would be sufficient to detect a moderate effect (*f* = .25; Cohen, 1992) with .80 power at *α* = .05. To accommodate increased variance expected in online data collection, a slightly smaller effect size (*f* = .22) was adopted, yielding a target of 165 participants per two-group design. Because Experiment 2 was designed to examine prediction updating separately for linearly and logarithmically spaced stimulus sets, sample size planning was conducted independently for each spacing condition. To further safeguard against data-quality losses, recruitment targeted 90 valid participants per condition.

During recruitment, 15 participants were excluded based on post-task questionnaire screening for use of prohibited strategies or substantial misunderstanding of the task (linear-long: 4; linear-short: 5; logarithmic-long: 3; logarithmic-short: 3). An additional five participants were excluded for having completed more than one condition, as identified by cross-checking questionnaire identifiers across conditions. These participants were replaced through continued recruitment. After recruitment was closed, one additional participant’s data could not be retrieved from the server; because this individual had not been formally rejected in the recruitment system, no replacement was recruited. Final sample sizes were therefore 90 participants in three conditions and 89 participants in one condition.

#### Experimental design

The experiment employed a between-subjects design with four parallel conditions: long-condition with linear spacing, short-condition with linear spacing, long-condition with logarithmic spacing, and short-condition with logarithmic spacing. Participants selected condition links without being informed of their corresponding group. In the long-condition groups, the longest comparison duration occurred more frequently during the skewed distribution phase; in the short-condition groups, the shortest comparison duration occurred more frequently.

#### Online implementation

All experimental events were controlled using Inquisit Web 6.4.2 (Millisecond Software, Seattle, WA). Participants completed the task on their own computers using the Inquisit Player application, and data were transmitted to a secure Millisecond server upon completion.

#### Temporal generalization task

**Fig. 7:**
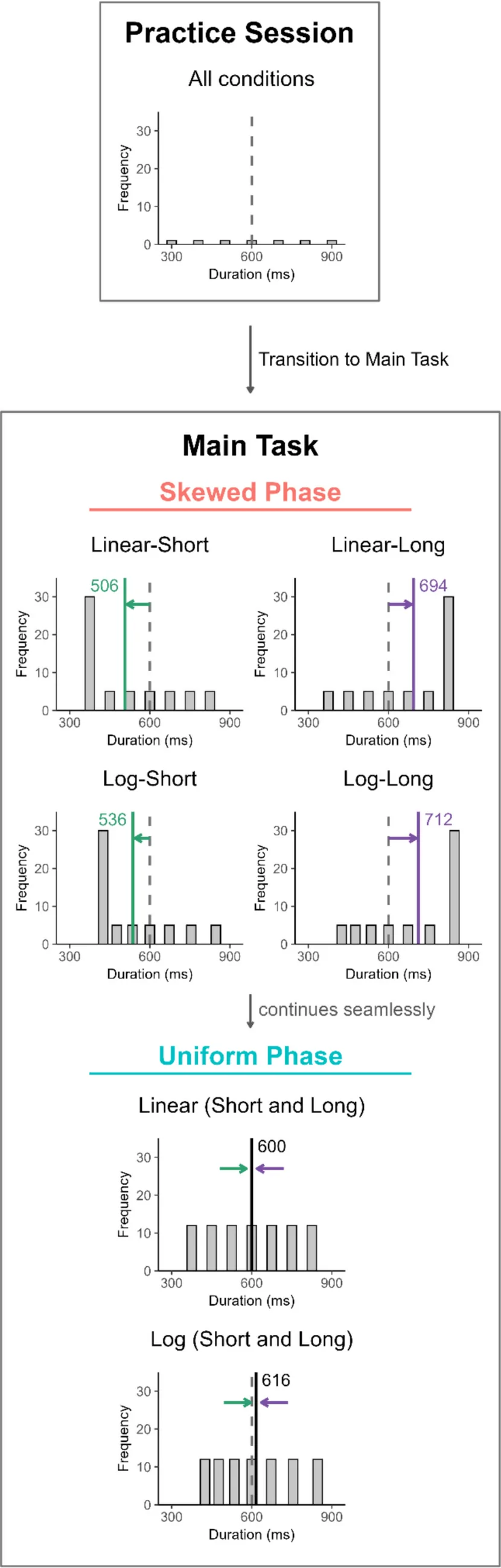
Experimental structure and stimulus distributions in Experiment 2. All participants completed the Practice Session for familiarization using a 600-ms standard duration and comparison durations ranging from 300 to 900 ms. In the Main Task, comparison stimuli were either linearly (Linear) or logarithmically (Log) spaced around a 600-ms standard duration. In the Skewed Phase, either the shortest (Short condition) or longest (Long condition) comparison stimulus was presented more frequently within each spacing condition. In the subsequent Uniform Phase, all comparison stimuli were presented equally often. Dashed vertical lines indicate the standard duration. Colored vertical lines (purple: Long condition; green: Short condition) indicate the weighted mean of the presented comparison-duration distributions in the Skewed Phase. In the Uniform Phase, black vertical lines indicate the weighted mean of the presented comparison-duration distributions. Colored arrows illustrate the expected directional shift of internal predictions toward reducing prediction error based on the stimulus distribution in each phase and condition

The overall task structure was identical to Experiment 1, including the use of 500-Hz pure tone stimuli, the 600-ms standard in the main task, initial standard presentations, the trial sequence, two successive phases (skewed distribution followed by uniform distribution), and the absence of feedback. The task structure and stimulus distributions across spacing conditions and experimental phases are illustrated in Fig. 7.

##### Stimuli

Comparison durations were either logarithmically spaced (425, 476, 535, 600, 673, 755, 847 ms) or linearly spaced (375, 450, 525, 600, 675, 750, 825 ms).

##### Response-key mapping

The “F” and “J” keys were used, and the assignment of the “same” response was counterbalanced across participants.

##### Pre-task instructions

Participants received detailed on-screen instructions specific to the online setting. They were instructed to complete the task in a quiet, distraction-free environment; to avoid using clocks, stopwatches, or any external timing aids; to base their judgments solely on the perceived duration of the auditory stimuli; and to refrain from using rhythmic movements, breathing, or other bodily strategies to measure duration, as well as from relying on non-temporal stimulus features (e.g., pitch, timbre, or background noise). Participants were informed of how to terminate the experiment if they chose to withdraw and were required to confirm that they had read and would follow these instructions before proceeding.

##### Practice session

Participants first adjusted stimulus volume to a comfortable level. They were then instructed to memorize the standard duration and judge whether each comparison duration was the same as the memorized standard. After one guided comparison trial, they completed the full practice procedure, consisting of standard presentations followed by seven judgment trials (one per comparison duration). Practice stimuli were 500-Hz pure tones with a 600-ms standard and comparison durations of 300, 400, 500, 600, 700, 800, and 900 ms. After the first guided trial, which used the 700-ms comparison duration, the message “It was not the same as the standard” was displayed to emphasize the need for careful discrimination. After the full set of seven practice trials, a second message appeared: “There was only one comparison that was the same as the standard.” This message was intended to emphasize that only the true standard should be endorsed as “same” and to encourage careful discrimination. Participants were then explicitly informed that both the standard and comparison durations used in the main task would differ from those used in the practice session, and that the relative frequency of comparisons matching the standard in the main task would also differ from that in practice. This instruction was intentionally phrased to discourage participants from relying on the specific durations encountered during the practice session. In fact, although the standard duration remained constant at 600 ms in both the practice session and the main task, the comparison durations differed between the practice session (300–900 ms in 100-ms steps) and the main task (375–825 ms in the linear condition; 425–847 ms in the logarithmic condition). No additional feedback was provided. Before the main task, participants were required to correctly identify the key assigned to the “same” response.

##### Instructions immediately before the main task

Immediately before the main task began, participants were reminded to minimize external distractions (e.g., to turn off smartphones), were informed of the approximate task duration (approximately 15 min), were instructed to complete the task continuously without breaks, and were told to respond carefully without emphasizing speed.

##### Post-task questionnaire

After completing the task, participants filled out a post-experiment questionnaire assessing how they memorized the standard duration and how they distinguished the comparison stimuli from the standard. These responses were used to verify comprehension of the instructions and to identify prohibited strategies or substantial misunderstanding. The questionnaire also included identifying information that was used to cross-check across conditions to identify duplicate participation.

#### Data processing and dependent measures

Data processing and dependent measures were identical to Experiment 1. Stimulus-wise proportions of “same” responses were computed separately for each participant and phase to construct temporal generalization gradients. Subjective standard value were calculated as same-response–weighted means of comparison durations. Gaussian functions were fitted to participant-level gradients in each phase, and the mean parameter (*μ*) was extracted. KDE was applied to *μ* values to visualize their distributions.

In addition to full-phase analyses, an exploratory block-level Gaussian analysis was conducted to examine early dynamics of reference updating. This analysis was performed to assess whether the early updating effects observed in Experiment 1 were also supported in Experiment 2, given its larger sample size. Block definitions and fitting procedures followed those of Experiment 1.

#### Data quality checks and exclusion criteria

Behavioral screening followed Experiment 1, with additional safeguards introduced due to the online setting. Participants producing unusually long response latencies (> 20 s) were excluded. Individual trials with latencies shorter than 150 ms were removed; participants for whom such trials constituted more than 10% of all trials were excluded from all analyses. To further screen for nonengagement, participants who used a single response key on more than 90% of trials were excluded.

As in Experiment 1, all analyses comparing skewed and uniform distribution phases included only paired data. Gaussian analyses included only participants whose fits converged successfully and whose parameter estimates fell within predefined plausible ranges. Unlike Experiment 1, additional goodness-of-fit criteria (*R*^*2*^ > .40) were applied not only to full-phase Gaussian fits but also to block-level Gaussian fits. As a result, final sample sizes differed across analyses and are summarized in Table 2.

**Table 2:** Sample sizes across analyses in Experiment 2

Analysis	N in long condition with linear spacing	N in short condition with linear spacing	N in long condition with logarithmic spacing	N in short condition with logarithmic spacing	Notes
Final analyzed sample	90	89	90	90	After recruitment-level screening
After behavioral screening	Skewed phase: 88 Uniform phase: 89	Skewed phase: 88 Uniform phase: 83	Skewed phase: 87 Uniform phase: 83	Skewed phase: 88 Uniform phase: 88	Latency > 20 s
Temporal generalization gradients (proportion of “same” responses)	87	83	82	87	Paired data only (skewed and uniform phases)
Subjective standard value	87	83	82	87	Paired data only (skewed and uniform phases)
Gaussian fitting (full-phase)	76	75	71	79	Successful Gaussian fits (paired data; 0 < *μ* < 1000 ms, *σ* < 500 ms, *R*^*2*^ > .40)
Exploratory block-level Gaussian fitting	21	16	20	20	Successful Gaussian fits (paired data; 0 < *μ* < 1000 ms, *σ* < 500 ms, *R*^*2*^ > .40)

#### Statistical analysis

Statistical analyses followed the general framework of Experiment 1 and were conducted in R. Stimulus-wise differences in the proportion of “same” responses between the skewed and uniform distribution phases were assessed using paired bootstrap resampling. Subjective standard value and Gaussian *μ* values estimated from the full phase were analyzed by using LMMs with Group and Phase as fixed effects and participant as a random intercept. Post hoc comparisons were performed using estimated marginal means with Bonferroni correction, and effect sizes were quantified using partial *R*^*2*^. In addition to these primary analyses, block-level Gaussian *μ* values were examined using bootstrap resampling of between-group differences at each block. Linearly and logarithmically spaced conditions were analyzed separately.

### Results

Experiment 2 examined whether the absence of reliable updating in the long-condition group observed in Experiment 1 reflected differences in prediction error magnitude arising from stimulus spacing, by directly comparing linearly and logarithmically spaced comparison durations. Accordingly, results are organized by condition (long vs. short), with linear and logarithmic spacing discussed adjacently within each condition.

#### Temporal generalization gradients: Distributional asymmetry

**Fig. 8:**
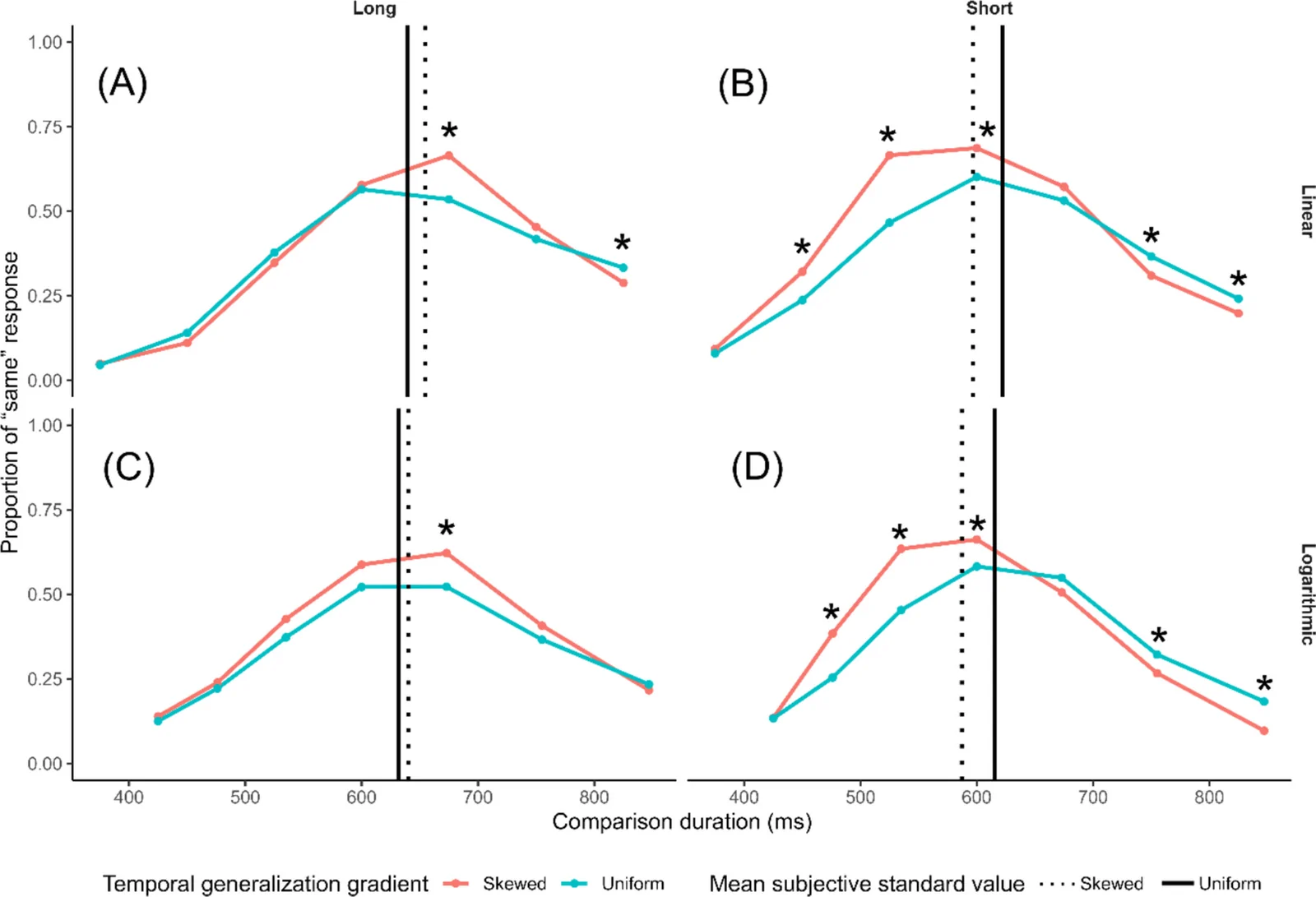
Temporal generalization gradients across phases and stimulus spacing in Experiment 2. (**A**) Linear–Long. (**B**) Linear–Short. (**C**) Logarithmic–Long. (**D**) Logarithmic–Short. Group mean proportions of “same” responses are shown as a function of comparison duration for the Skewed and Uniform phases. Asterisks indicate statistically significant phase differences at corresponding comparison durations (*p* < .05). Dotted and solid vertical lines represent the group mean subjective standard values (SSVs) in the Skewed and Uniform phases, respectively

Figure 8 summarizes the group-mean temporal generalization gradients across phases. Vertical lines indicate the corresponding mean SSVs.

##### Long-condition group

With linearly spaced stimuli (*n* = 87), bootstrap resampling revealed a statistically reliable reduction in the proportion of “same” responses in the uniform distribution phase at the 675-ms comparison stimulus (*estimate* =  − 0.130, 95% CI [− 0.205, − 0.056]). An additional reliable increase was observed at 825 ms (*estimate* = 0.045, 95% CI [0.001, 0.088]). No other comparison durations showed statistically reliable phase differences.

With logarithmically spaced stimuli (*n* = 82), a statistically reliable reduction in “same” responses was again observed at 675 ms (*estimate* =  − 0.100, 95% CI [− 0.170, − 0.032]), together with a smaller but reliable reduction at the 600-ms standard (*estimate* =  − 0.066, 95% CI [− 0.125, − 0.008]).

##### Short-condition group

In contrast, the short condition showed phase-dependent changes across multiple comparison durations. With linearly spaced stimuli (*n* = 83), statistically reliable reductions in “same” responses in the uniform distribution phase were observed for several durations shorter than the standard (e.g., 450 ms: *estimate* =  − 0.084, 95% CI [− 0.146, − 0.023]; 525 ms: *estimate* =  − 0.199, 95% CI [− 0.259, − 0.139]) and at the standard itself (600 ms: *estimate* =  − 0.085, 95% CI [− 0.157, − 0.015]). Conversely, “same” responses were reliably higher for longer-than-standard stimuli (e.g., 750 ms: *estimate* = 0.057, 95% CI [0.004, 0.110]; 825 ms: *estimate* = 0.043, 95% CI [0.006, 0.083]).

With logarithmically spaced stimuli (*n* = 87), a similar pattern was observed: “same” responses in the uniform phase were significantly lower for shorter-than-standard durations (e.g., 476 ms: *estimate* =  − 0.130, 95% CI [− 0.193, − 0.068]; 535 ms: *estimate* =  − 0.181, 95% CI [− 0.246, − 0.117]) and higher for longer-than-standard durations (e.g., 755 ms: *estimate* = 0.055, 95% CI [0.002, 0.109]; 847 ms: *estimate* = 0.086, 95% CI [0.046, 0.131]).

Taken together, these gradient-level results show robust phase-dependent changes in distributional asymmetry in the short condition and more localized changes in the long condition, without a clear dissociation between linear and logarithmic spacing.

#### Subjective standard value

For the long-condition group, mean SSVs (± *SD*) were 654.91 ms (76.63) and 639.66 ms (79.17) in the skewed and uniform distribution phases, respectively, with linearly spaced stimuli (*n* = 87), and 640.00 ms (80.23) and 631.73 ms (80.23) with logarithmically spaced stimuli (*n* = 82). For the short-condition group, the corresponding mean SSVs were 596.55 ms (78.78) and 622.03 ms (78.50) with linear spacing (*n* = 83), and 587.07 ms (76.25) and 615.30 ms (75.26) with logarithmic spacing (*n* = 87). The corresponding mean SSVs are illustrated by the vertical lines in Fig. 8.

Subjective standard value were analyzed by using a LMM with Group (long vs. short) and Phase (skewed vs. uniform) as fixed effects and participant as a random intercept. Under both linear and logarithmic spacing, the model revealed significant main effects of Group (linear: *F*(1,168) = 10.68, *p* = .001; logarithmic: *F*(1,167) = 9.13, *p* = .003) and significant Group × Phase interactions (linear: *F*(1,168) = 40.97,* p* < .001; logarithmic: *F*(1,167) = 28.95, *p* < .001); the main effect of Phase was significant under logarithmic spacing (*F*(1,167) = 8.66, *p* = .004) but not under linear spacing (*F*(1,168) = 2.59, *p* = .11). In both spacing conditions, the interaction accounted for the largest proportion of unique variance (linear: partial *R*^*2*^ = .196, 95% CI [.103, .304]; logarithmic: partial* R*^*2*^ = .148, 95% CI [.064, .252]), and the fixed-effects models explained 19% (linear) and 17.3% (logarithmic) of the variance in SSV.

**Fig. 9:**
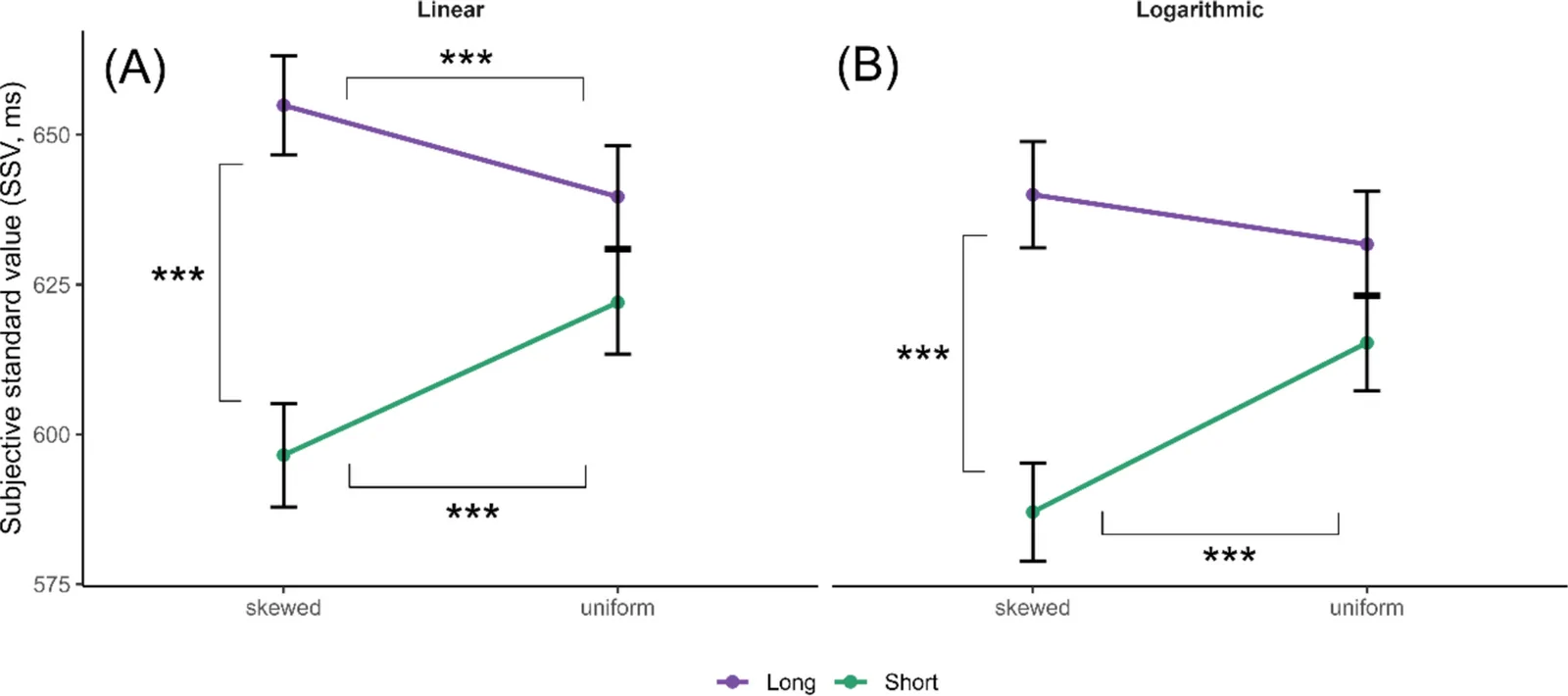
Phase-dependent changes in subjective standard values (SSVs) in Experiment 2. (**A**) Linear spacing. (**B**) Logarithmic spacing. Group mean subjective standard values (SSVs) are shown for the skewed and uniform distribution phases in the Long and Short conditions. Results are based on linear mixed-effects models. Error bars represent ± 1 *SE*. Asterisks indicate statistically significant contrasts (***p < .001)

Post hoc comparisons (skewed − uniform) showed that in the long-condition group, SSV decreased significantly under linear spacing (*estimate* = 15.2 ms, *SE* = 4.45, *p* = .0008) but not under logarithmic spacing (*estimate* = 8.27 ms, *SE* = 4.87, *p* = .0911), whereas in the short-condition group, SSV increased significantly from the skewed to the uniform distribution phase under both linear and logarithmic spacing (*estimate* =  − 25.5 ms, *SE* = 4.55, *p* < .0001; *estimate* =  − 28.23 ms, *SE* = 4.73, *p* < .0001). During the skewed distribution phase, SSVs were significantly higher in the long- than in the short-condition group under both spacing types (*estimate* = 58.4 ms, *SE* = 12.1, *p* < .0001; *estimate* = 52.9 ms, *SE* = 12.0, *p* < .0001), whereas this group difference was not reliable in the uniform distribution phase under either linear or logarithmic spacing (*estimate* = 17.6 ms, *SE* = 12.1, *p* = .15; *estimate* = 16.4 ms, *SE* = 12.0, *p* = .1715). Figure 9 presents the LMM results for SSV.

#### Gaussian fit for generalization gradients: Peak shift

For the long-condition group, mean Gaussian *μ* values (± *SD*) were 661.91 ms (89.02) and 646.13 ms (89.02) in the skewed and uniform distribution phases, respectively, with linearly spaced stimuli (*n* = 76), and 642.74 ms (85.04) and 638.98 ms (85.04) with logarithmically spaced stimuli (*n* = 71). For the short-condition group, the corresponding mean *μ* values were 593.62 ms (89.02) and 625.34 ms (89.02) with linear spacing (*n* = 75), and 583.68 ms (85.04) and 613.66 ms (85.04) with logarithmic spacing (*n* = 79).

**Fig. 10:**
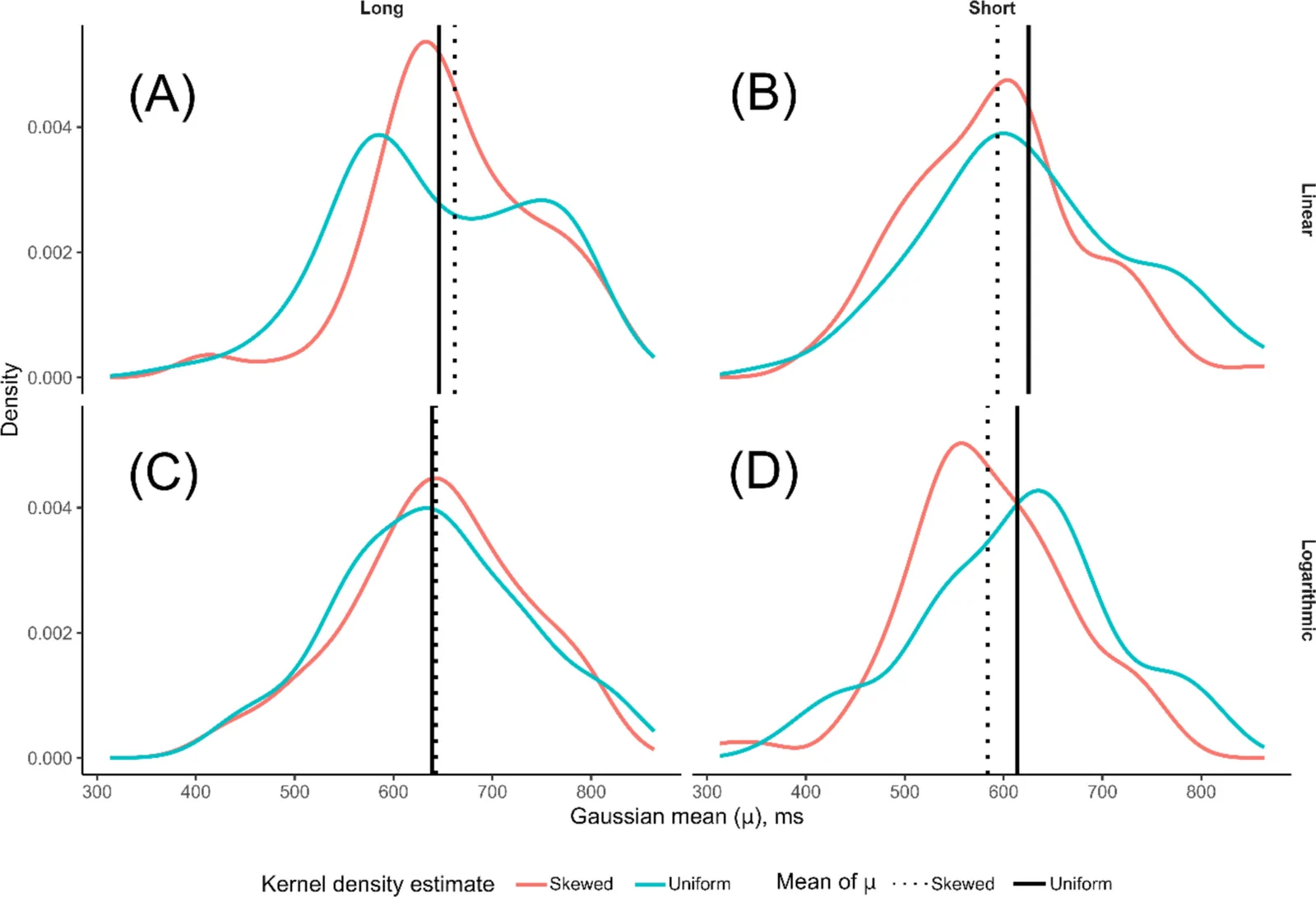
Distribution of participant-level Gaussian *μ* estimates across phases in Experiment 2. (**A**) Linear–Long. (**B**) Linear–Short. (**C**) Logarithmic–Long. (**D**) Logarithmic–Short. Kernel density estimates depict the distribution of Gaussian mean (*μ*) values obtained by fitting Gaussian functions to each participant’s temporal generalization gradients in the Skewed and Uniform phases. Dotted and solid vertical lines represent the phase-wise mean *μ* values in the Skewed and Uniform phases, respectively

Figure 10 shows the distributions of participant-level Gaussian *μ* estimates across phases, with vertical lines indicating the phase-wise mean *μ*. In the long-condition group, spacing-dependent differences were evident: under linear spacing, the uniform-phase distribution was more dispersed, with one concentration near the standard (~ 600 ms) and another toward longer durations, indicating heterogeneous peak locations across participants, whereas under logarithmic spacing, *μ* distributions overlapped substantially across phases. In contrast, in the short-condition group, *μ* distributions shifted coherently toward longer durations from the skewed to the uniform phase under both spacing conditions.

Gaussian *μ* values were analyzed using a LMM with Group (long vs. short) and Phase (skewed vs. uniform) as fixed effects and participant as a random intercept. Under both linear and logarithmic spacing, the model revealed significant main effects of Group and Phase, as well as significant Group × Phase interactions. Under linear spacing, significant effects were observed for Group, *F*(1,149) = 8.79, *p* = .0035, Phase, *F*(1,149) = 3.96, *p* = .048, and their interaction, *F*(1,149) = 35.31, *p* < .001. Under logarithmic spacing, significant effects were likewise found for Group, *F*(1,148) = 8.41, *p* = .0043, Phase, *F*(1,148) = 9.35, *p* = .0027, and the Group × Phase interaction, *F*(1,148) = 15.48, *p* < .001. In both spacing conditions, the interaction accounted for the largest proportion of unique variance (linear: partial *R*^*2*^ = .192, 95% CI [.094, .307]; logarithmic: partial *R*^*2*^ = .095, 95% CI [.025, .197]), and the fixed-effects models explained 19.1% (linear) and 14.6% (logarithmic) of the variance in *μ*.

**Fig. 11:**
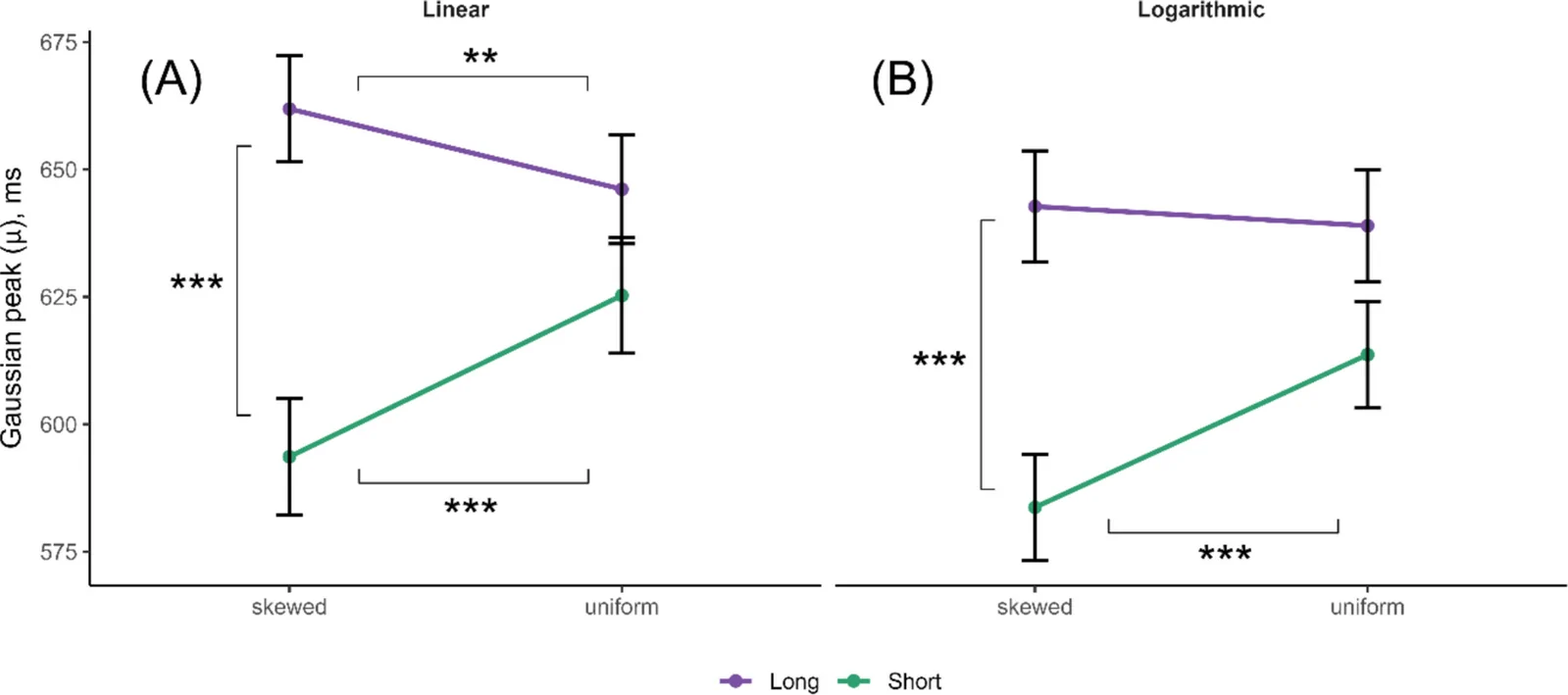
Phase-dependent shifts in Gaussian peak location (*μ*) in Experiment 2. (**A**) Linear spacing. (**B**) Logarithmic spacing. Group mean Gaussian peak locations (*μ*) are shown for the skewed and uniform distribution phases in the Long and Short conditions. Results are based on linear mixed-effects models. Error bars represent ± 1 *SE*. Asterisks indicate statistically significant contrasts (**p < .01; ***p < .001)

Post hoc comparisons (skewed − uniform) showed that in the long-condition group, *μ* decreased significantly under linear spacing (*estimate* = 15.8 ms, *SE* = 5.63, *p* = .0057) but not under logarithmic spacing (*estimate* = 3.76 ms, *SE* = 6.22, *p* = .55), whereas in the short-condition group, *μ* increased significantly from the skewed to the uniform distribution phase under both spacing conditions (linear: *estimate* =  − 31.7 ms, *SE* = 5.67, *p* < .0001; logarithmic: *estimate* =  − 29.98 ms, *SE* = 5.90, *p* < .0001). During the skewed distribution phase, *μ* values were significantly higher in the long- than in the short-condition group under both linear and logarithmic spacing (linear: *estimate* = 68.3 ms, *SE* = 15.6, *p* < .0001; logarithmic: *estimate* = 59.1 ms, *SE* = 15.2, *p* = .0001), whereas this group difference was not reliable in the uniform distribution phase under either spacing condition (linear: *estimate* = 20.8 ms, *SE* = 15.6, *p* = .18; logarithmic: *estimate* = 25.3 ms, *SE* = 15.2, *p* = .097). Figure 11 presents the LMM results forμ.

Across spacing conditions, the mixed-effects analyses of Gaussian *μ* reproduced the pattern observed for SSV, including the critical dissociation in the long-condition group: reliable downward shifts in *μ* were observed under linear spacing but not under logarithmic spacing, whereas the short-condition group showed robust upward shifts under both spacing conditions. Together with the distributional patterns shown in Fig. 10, these results indicate that spacing-dependent asymmetry is expressed primarily in the long-condition group. Attenuation of updating was observed only under logarithmic spacing.

#### Exploratory block-level Gaussian fit: Early reference updating

To assess whether directional updating emerged early in learning, Gaussian peak locations (*μ*) were estimated for successive blocks and compared between groups (long − short) separately for linear and logarithmic spacing. This exploratory analysis was conducted on participants whose Gaussian fits converged across blocks and is intended to characterize temporal patterns rather than provide confirmatory tests.

Under linear spacing, no reliable group difference was observed in the first block of the skewed phase, but a clear separation emerged from the second block and remained evident in the third block. Under logarithmic spacing, by contrast, a reliable group difference was already present in the first skewed block remained evident in the second block, and attenuated in the third block.

In the uniform distribution phase, between-group differences were no longer reliable across later blocks for either spacing type, and point estimates decreased over time, consistent with convergence toward a common reference value.

**Fig. 12:**
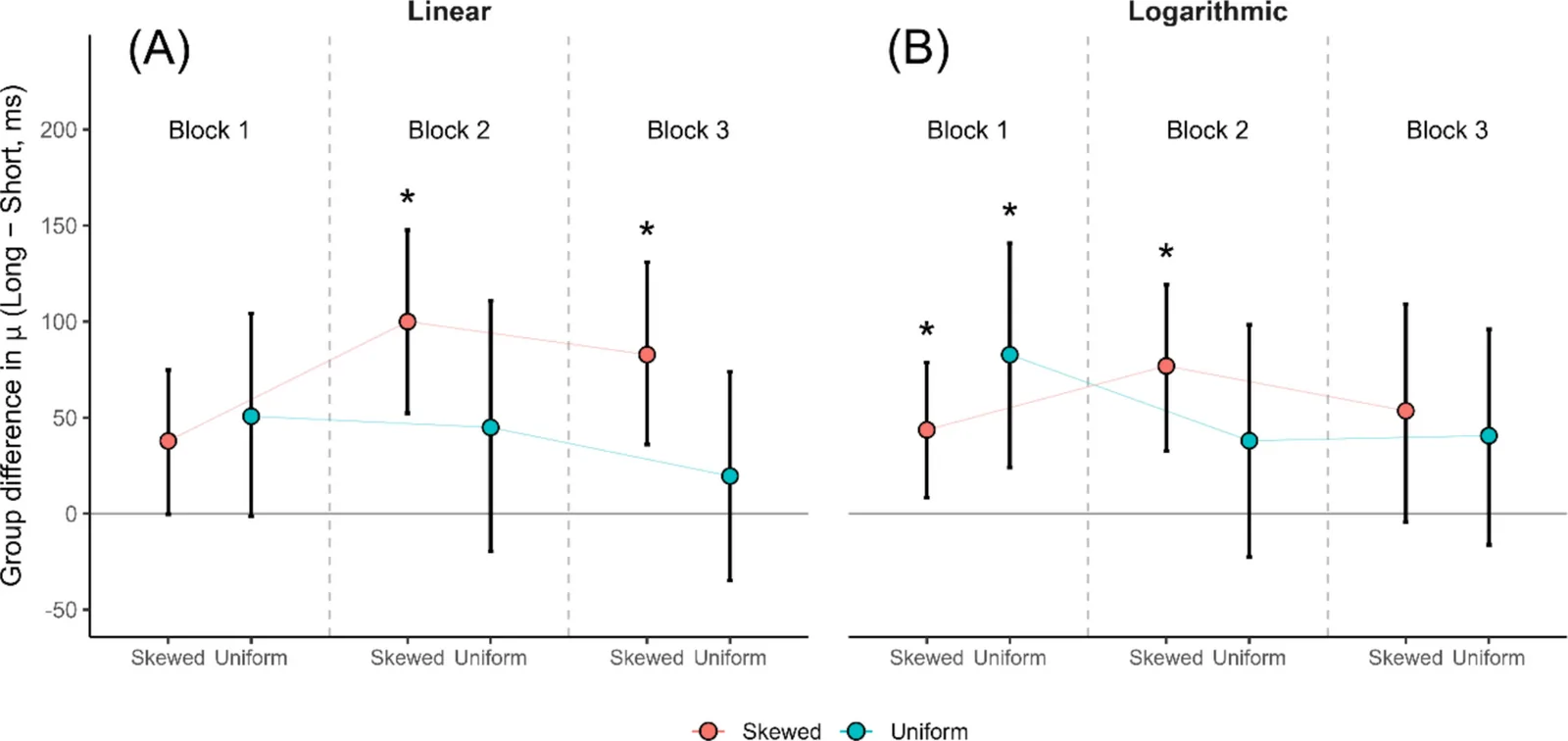
Block-level group differences in Gaussian peak location (*μ*) in Experiment 2. (**A**) Linear spacing. (**B**) Logarithmic spacing. Bootstrapped estimates of the group difference in Gaussian *μ* (Long − Short) are shown separately for the skewed and uniform phases within each block. Error bars indicate 95% bootstrap confidence intervals. The gray horizontal solid line marks zero difference. Asterisks indicate intervals that do not include zero, reflecting statistically reliable group differences. Under linear spacing, the temporal profile of group differences across the skewed and uniform phases was broadly consistent with directional divergence followed by convergence. Under logarithmic spacing, however, attenuation of the group difference during the skewed phase suggested a less sustained divergence between groups

Together, these exploratory results indicate that reference updating emerged early during both phases, with distinct temporal profiles under linear and logarithmic spacing. Block-level group differences in Gaussian peak location are shown in Fig. 12.

### Discussion

Experiment 2 tested the hypothesis that the attenuated reference updating observed in the long-condition group in Experiment 1 reflected insufficient prediction error arising from logarithmically spaced stimuli. By directly comparing linearly and logarithmically spaced comparison sets, the experiment assessed whether increasing prediction error in the shorter direction would destabilize the long-shifted reference. Across converging indices—mean SSVs, Gaussian peak locations (*μ*), and population-level kernel density estimates—the results consistently supported this hypothesis. Reliable across-phase updating in the long-condition group was observed under linear spacing (larger prediction error) but not under logarithmic spacing (smaller prediction error). Thus, Experiment 2 provides direct evidence that reference updating in the long condition depends on the magnitude of prediction error as shaped by stimulus structure, rather than reflecting a general limitation of the updating mechanism.

The kernel density estimates of Gaussian *μ* clarified how reference updating was implemented across participants in the long-condition group. Under logarithmic spacing, *μ* distributions showed minimal change across phases, whereas under linear spacing, heterogeneous changes were observed, with concentrations both near the original standard and toward longer durations. This pattern suggests that the group-level mean shift under linear spacing reflected selective updating in a subset of participants, while others preserved the long-shifted reference established during the skewed phase. Thus, when prediction error in the shorter direction was larger (linear spacing), the long-shifted reference was destabilized in some participants, whereas when prediction error was structurally smaller (logarithmic spacing), comparable destabilization was not observed. Importantly, whereas the mixed-effects analyses captured the systematic directional tendency at the group level, the KDEs revealed the inter-individual diversity underlying this effect.

In interpreting these findings, it is important to consider the properties of the indices used. Both SSVs and Gaussian *μ* reflect reference updating as distributional asymmetry or peak displacement in the temporal generalization gradients; however, temporal generalization is intrinsically asymmetric, such that longer comparison durations are more likely to be judged as “the same,” particularly with linearly spaced stimulus sets (Wearden, 1992; Wearden & Towse, 1994). This task-intrinsic asymmetry likely contributed to the elevated reference values observed across phases. Nevertheless, Experiment 2 also revealed effects running counter to this intrinsic tendency: in the long-condition group, reliable shifts toward shorter values were observed under linear spacing, indicating downward updating despite the inherent bias toward longer durations. These results therefore indicate that reference updating follows the direction of prediction error, rather than merely reflecting response asymmetries, consistent with the prediction error minimization hypothesis.

Finally, block-level analyses of Gaussian *μ* were conducted to descriptively examine early within-phase dynamics. Consistent with Experiment 1, between-group differences emerged early under skewed distributions and diminished under the uniform distribution. Although some spacing-dependent differences were observed, these analyses were exploratory and are not central to the conclusions of Experiment 2. Accordingly, the primary evidence for spacing-dependent updating rests on the converging results from SSVs, full-phase Gaussian *μ*, and the population-level KDEs.

### General discussion

This study examined whether prediction updating in time perception follows the principle of prediction error minimization. Across two experiments, we manipulated the direction and magnitude of prediction error in a temporal generalization task and examined the direction of internal temporal reference updating as a function of these manipulations. Consistent with our hypothesis, temporal predictions were updated in the direction that reduced systematic prediction error, and updating was observed only when prediction error reached sufficient magnitude. Together, these findings provide a direct behavioral test of the prediction error minimization hypothesis in sub-second time perception, demonstrating that internal temporal references are updated in a principled manner that reduces prediction error.

A related methodological consideration concerns the total number of trials. The present task was intentionally compact to allow temporal reference updating within a continuous, uninterrupted context. The internal temporal reference may reflect memory for the standard, an implicitly computed contextual statistic, or both. In either case, updating requires that predictions be maintained within a coherent temporal context, which may be disrupted in extended tasks by fatigue, strategy shifts, or segmentation of experience. Trial numbers were therefore chosen to balance sensitivity to distributional learning with preservation of a continuous reference. This design enabled detection not only of reliable updating, but also of conditions under which updating failed to occur, as well as sensitivity to prediction error magnitude and inter-individual heterogeneity. Future studies that deliberately extend the temporal scale of the task may provide a principled way to dissociate memory-based and context-based accounts of the temporal reference.

An additional implication of the present design concerns the role of motor involvement in reference updating. The present study did not aim to evaluate motor contributions to temporal context effects directly. However, because this study featured a temporal generalization task that does not require motor involvement during the judgment process, the present findings may be interpreted as indicating that reference updating can occur even when temporal judgments are made independently of overt motor responses. In this respect, the present findings may be somewhat more consistent with studies examining temporal context effects at the perceptual level (Zimmermann & Cicchini, 2020) than with findings emphasizing motor-response specificity of temporal prior generalization (Roach et al., 2017).

Although this study was designed with minimal theoretical assumptions to assess the direction of prediction updating, the findings raise a deeper question: whether prediction error and the extent of updating are linearly related. In Experiment 2, larger prediction errors were associated with smaller magnitudes of updating. During the skewed distribution phase, both subjective standard values and Gaussian μ estimates remained closer to the original standard under conditions involving larger prediction error, in both long- and short-condition groups, indicating that updating did not scale proportionally with prediction error.

Rather, the results are consistent with a non-linear relationship, in which extremely large prediction errors exert reduced influence or even produce contrastive effects. Sherif et al. (1958) showed that anchors elicit assimilation when relatively close to the stimulus range, but contrast when they deviate too far. A similar mechanism may have operated here: as prediction error increases beyond an effective range, its influence on reference updating may diminish. At the same time, Experiment 2 also showed that insufficient prediction error failed to drive updating. Together, these findings suggest that the relationship between prediction error and updating may follow a bounded, non-linear function, potentially resembling an inverted-U. While this interpretation remains speculative, future studies should systematically manipulate prediction error magnitude to test its influence on the direction and extent of prediction updating.

Despite these theoretical implications, several limitations constrain interpretation. First, we did not directly examine the role of precision in moderating prediction updating. Predictive processing accounts posit that prediction errors are weighted by their relative precision (Feldman & Friston, 2010; Friston, 2009); however, the present design did not manipulate or independently assess uncertainty in either temporal expectations or sensory inputs. Future studies should test whether the precision of temporal priors or sensory encoding modulates the direction and extent of prediction updating. Second, our primary analyses relied on indices aggregated across the skewed and uniform phases, limiting insight into how predictions evolved on a finer timescale. Trial-level modeling approaches or adaptive experimental designs may better capture the dynamics of belief updating and the temporal evolution of internal reference representations. Third, temporal generalization tasks introduce systematic response asymmetries, such that longer comparison durations are more likely to be judged as “the same” than shorter ones. Although Experiment 2 demonstrated that reference updating can occur against this intrinsic tendency, such asymmetries nonetheless constrain how shifts in summary indices can be interpreted.

This task-intrinsic asymmetry also suggests a concrete methodological direction for future work. Because summary indices from temporal generalization gradients are estimated on top of an upward response bias, apparent shifts in the internal reference may partly reflect the direction and magnitude of this bias. One way to directly assess its contribution would be to reverse the order of the experimental phases, such that a uniform distribution precedes a skewed distribution. Under this design, the same asymmetry should exaggerate apparent shifts in the long-condition group and attenuate them in the short-condition group. Comparing the present design with such a phase-reversed design would allow estimation of the task-intrinsic bias and provide a principled means to dissociate genuine reference updating from response asymmetries, enabling more precise modeling of temporal prediction updating.

Beyond the transient predictions engaged by the temporal generalization task, a broader predictive architecture implies that the brain maintains multiple predictions, or “internal references” (Bausenhart et al., 2016), formed across different timescales. Glasauer and Shi (2021) showed that central tendency bias is reduced when stimuli follow a random-walk sequence rather than a randomized one, suggesting that the brain predicts not only stimulus values but also their patterns of change. When stimuli follow gradual transitions, they align with longer-term expectations and elicit minimal prediction error. In contrast, randomly sequenced stimuli violate these expectations and produce errors that trigger belief updating or perceptual bias. These findings underscore that prediction is not merely a transient construct formed within an experimental context but is shaped by deeply embedded and often implicit expectations. Understanding how long-term, layered predictions interact with task-level manipulations may therefore offer deeper insight into the flexibility and constraints of the predictive mechanisms in time perception. Human time perception lacks the precision and stability of a physical clock; rather than reflecting a limitation, this departure from clock-like accuracy may be fundamental to its capacity to support adaptive behavior in a highly diverse and continuously changing environment. A simple organizing principle of brain function—prediction error minimization—may underlie this competence of time perception.

Although predictive processing continues to generate debate, with mixed empirical support for its central claims (Walsh et al., 2020), it remains a powerful and influential framework across cognitive, behavioral, and neural sciences. When carefully applied, it offers a valuable foundation for experimental research on the brain’s predictive nature, particularly within time perception.

## Data Availability

Data or materials for the experiments are available upon request.

## Code availability

Codes for the experiments or analyses are available upon request.

## Declarations

### Open practices statements

Data or materials for the experiments are available upon request, and no experiments were preregistered.
